# *Pseudodiaptomus marinus* Sato, 1913 in the Black Sea: morphology, genetic analysis, and variability in seasonal and interannual abundance

**DOI:** 10.7717/peerj.10153

**Published:** 2020-10-22

**Authors:** Alexandra Gubanova, Inna Drapun, Oksana Garbazey, Olga Krivenko, Ekaterina Vodiasova

**Affiliations:** 1 Plankton department, A.O. Kovalevsky Institute of Biology of the Southern Seas of RAS, Sevastopol, Russia; 2 Marine biodiversity and functional genomics laboratory, A.O. Kovalevsky Institute of Biology of the Southern Seas of RAS, Sevastopol, Russia

**Keywords:** *Pseudodiaptomus marinus*, Non-indigenous species, Black Sea, Seasonal dynamics, Sequence analysis, cytb, rRNA, Morphology

## Abstract

Calanoid copepod *Peudodiaptomus marinus* Sato, 1913 was first recorded in Sevastopol Bay in the northern Black Sea in September 2016. We performed regular observations of this new invasive species between October 2016 and December 2018. We conducted bi-weekly plankton sampling at three stations located within or adjacent to Sevastopol Bay. This is the first paper to combine (i) a detailed morphological study, (ii) molecular genetic analysis, and (iii) an investigation of *P. marinus* seasonal dynamics and interannual abundance variability in the coastal Black Sea. Our morphological research showed similarities between *Pseudodiaptomus* specimens and existing *P. marinus* illustrations and descriptions. Our morphological analysis results were confirmed using molecular genetic studies. Based on the genetic variability of ITS2 and *cytb*, we found that all investigated specimens from Sevastopol Bay belonged to *P. marinus.* Investigations of *P. marinus* seasonal and interannual abundance variability showed the same seasonal patterns throughout the studied period, with a higher seasonal abundance from October to November and one pronounced density peak in autumn. The highest abundances (2,000 ind m^–3^ at the mouth of the bay and more than 5,000 ind m^–3^at its centre) were recorded in November 2018. In the samples, we found adults of both sexes, including ovigerous females, copepodites I–V, and nauplii, suggesting that the species reproduce in Sevastopol Bay. Our research indicated that *P. marinus* is a new non-indigenous species (NIS) in the Black Sea, and we will discuss a possible vector of its introduction into this basin.

## Introduction

Over 30 years ago, marine biological invasions were identified as one of the most serious global environmental changes (Carlton, 1999; Occhipinti-Ambrogi & Savini, 2003). Since then, the list of non-indigenous species (NIS) in different parts of the World Ocean has grown, altering an increasing number of marine ecosystems (Chan & Briski, 2017; Pooley & Queiroz, 2018), and expanding the scope of challenges caused by these alien species (Simberloff et al., 2013; Katsanevakis et al., 2014).

Semi-enclosed basins such as the Black Sea are especially sensitive to the impact of anthropogenic factors, including invading alien species. The introduction of the zooplanktivorous comb jelly *Mnemiopsis leidyi* into the Black Sea was one of the most catastrophic invasions to date (Boxshall, 2007). The explosive growth of *M. leidyi* in the 1990s triggered a number of changes in the entire basin that are still ongoing (Shiganova et al., 2001; Gubanova et al., 2001; Kideys, 2002; Finenko & Romanova, 2000; Oguz, Fach & Salihoglu, 2008). In the 1990s, two native copepods, *Oithona nana* and *Acartia margalefi*, disappeared completely from the Black Sea, resulting in a decrease in the total abundance of copepods by more than one order of magnitude (Gubanova et al., 2001). *O. nana* was a dominant species in the coastal area during the summer-autumn season, and was crucial in supporting the equilibrium of the pelagic coastal ecosystems. After the introduction and spread of the ctenophore *Beroe ovata,* which feeds on *M. leidyi*, predatory pressure on Black Sea zooplankton decreased in the early 2000s (Finenko et al., 2006) and another non-indigenous species, the copepod *Oithona davisae*, appeared in the Black Sea (Gubanova & Altukhov, 2007). *O. davisae* became dominant during the summer-autumn season in the coastal area, occupying the niche of the now-disappeared and native *O. nana* (Altukhov, Gubanova & Mukhanov, 2014). This new invasion caused serious changes for the zooplankton and the ecosystem as a whole (Gubanova et al., 2019).

*P. marinus* Sato, 1913 is a copepod species that was recently introduced to the Black Sea. It was initially discovered during routine plankton surveys of Sevastopol Bay in September 2016 (Garbazey et al., 2016). This estuarine-coastal species is believed to have originated from the Northwestern Pacific (Walter, 1987; Walter et al., 2002) and was first recorded near the coast of Hokkaido in Northern Japan (Sato, 1913). *P. marinus* was recorded in new areas of the Pacific Ocean since the 1940s (Brodskii, 1948; Razouls et al., 2005–2020;–ref in Brylinski et al. 2012). Since 2007, the species has rapidly expanded into European waters (De Olazabal & Tirelli, 2011; Sabia et al., 2017; Uttieri et al, 2020). The first appearance of *P. marinus* was reported to be in the Gulf of Izmir, Aegean Sea, Turkey (S Besiktepe, pers. comm., 2020). Currently, *P. marinus* is one of the most widespread copepods in the World Ocean. A number of studies have revealed *P. marinus*’s invasion history and specific biological and behavioural traits that demonstrate its ability to successfully adapt to new environments (Brylinski et al., 2012; Sabia et al., 2017; Uttieri et al., 2020).

More than 80 species of the *Pseudodiaptomus* genus are known worldwide (Razouls et al., 2005–2020). Many of them have subtle morphological differences, which can make it difficult to identify species of the genus. In Sato’s (1913) original description of *P. marinus*, clear drawings and descriptions of all morphological features were not provided.

Subsequently, Grindley & Grice (1969) presented their complete morphological description based on specimens collected in Port Louis Harbour, Mauritius. The authors pointed out differences across the morphologies of Mauritian, Hawaiian, and Japanese specimens, as well as from previously published descriptions of *P. marinus.* They determined that the Mauritian population may have been represented by ecophenotypes (Grindley & Grice, 1969). *Walter (1986)* concluded that the Mauritian specimens should be considered a separate species, *Pseudodiaptomus* cf. *marinus*. Fleminger & Kramer (1988) agreed with this view and noted the similarities between this species and *Pseudodiaptomus philippinensis* Walter, 1986.

The integration of classical morphology and molecular approaches has provided new prospects for solving taxonomic challenges and other complicated aspects of NIS biology and ecology (Sabia et al., 2017; Uttieri et al., 2020). Nevertheless, molecular analysis has been carried out on only a small number of *P. marinus* specimens from only a few points in the World Ocean, which has limited the discussion of its dispersal pathways and possible origins (Albaina et al., 2016; Uttieri et al., 2020).

For species identification, several DNA markers can be used such as 18S, 28S or ITS of rRNA genes, cytochrome oxidase I (COI) or cytochrome b (*cytb*) of mtDNA genes. COI is one of the most widespread DNA markers in invertebrates, but it can cause some difficulties in calanoid copepods. COI’s small amount of mtDNA and high evolutionary rate are its main challenges, as they can cause mutations at the annealing sites of universal primers. Its overall success rate for amplification and sequencing is only 31% (Hirai, Shimode & Tsuda, 2013; Bradford-Grieve, Blanco-Bercial & Boxshall, 2017; Rocha et al., 2018).

ITS1 and ITS2 rDNA have demonstrated better sequencing and amplification rates than COI for calanoid copepods. Moreover, the amount of DNA is not important because ITS is a multicopy gene (Naidoo et al., 2013; Sabia et al., 2017). The success rate for intrageneric discrimination using ITS2 for *Pseudodiaptomus* species is higher than 90% (Rocha et al., 2018). To date, the NCBI has abundant data on the phylogenetic reconstruction of Calanoida (Copepoda) based on ITS2 variability. Moreover, the primers for fragments of mitochondrial gene *cytb*, designed for molluscan taxa, were applied for *P. marinus* (Ohtsuka et al., 2018).

In 2018, Dr. Marco Utierri and scientists from nine European countries established the working group (WG) titled “Towards a EURopean OBservatory of the non-indigenous calanoid copepod *P. marinus*” (EUROBUS). This WG aimed to create a European network of institutions and researchers working on various aspects of *P. marinus’* biology and ecology. This included conducting molecular genetic investigations of populations from different localities in order to better understand NIS introduction vectors, dispersal pathways, and the possible consequences (threats or opportunities) of their spread and establishment in European basins. The main agenda for 2020 included recording detailed morphological descriptions, comparing specimens’ diagnostic characters from different areas, and performing comparative analysis of the *P. marinus*’ seasonality at different sites in European waters.

Our investigation fully abided by the EUROBUS WG objectives and provides the following:

(i) a detailed description of the main distinctive characteristics of *P. marinus* specimens from Sevastopol Bay to use as the basis for further morphological research and comparisons with individuals from other biotopes,

(ii) a genetic analysis to identify *P. marinus’* taxonomy and possible dispersal pathways into Sevastopol Bay, and

(iii) observations of the seasonal and interannual variations in *P. marinus* abundance during the studied period in the coastal area of the Black Sea.

## Materials & Methods

### Sample collection and zooplankton processing

**Figure 1:**
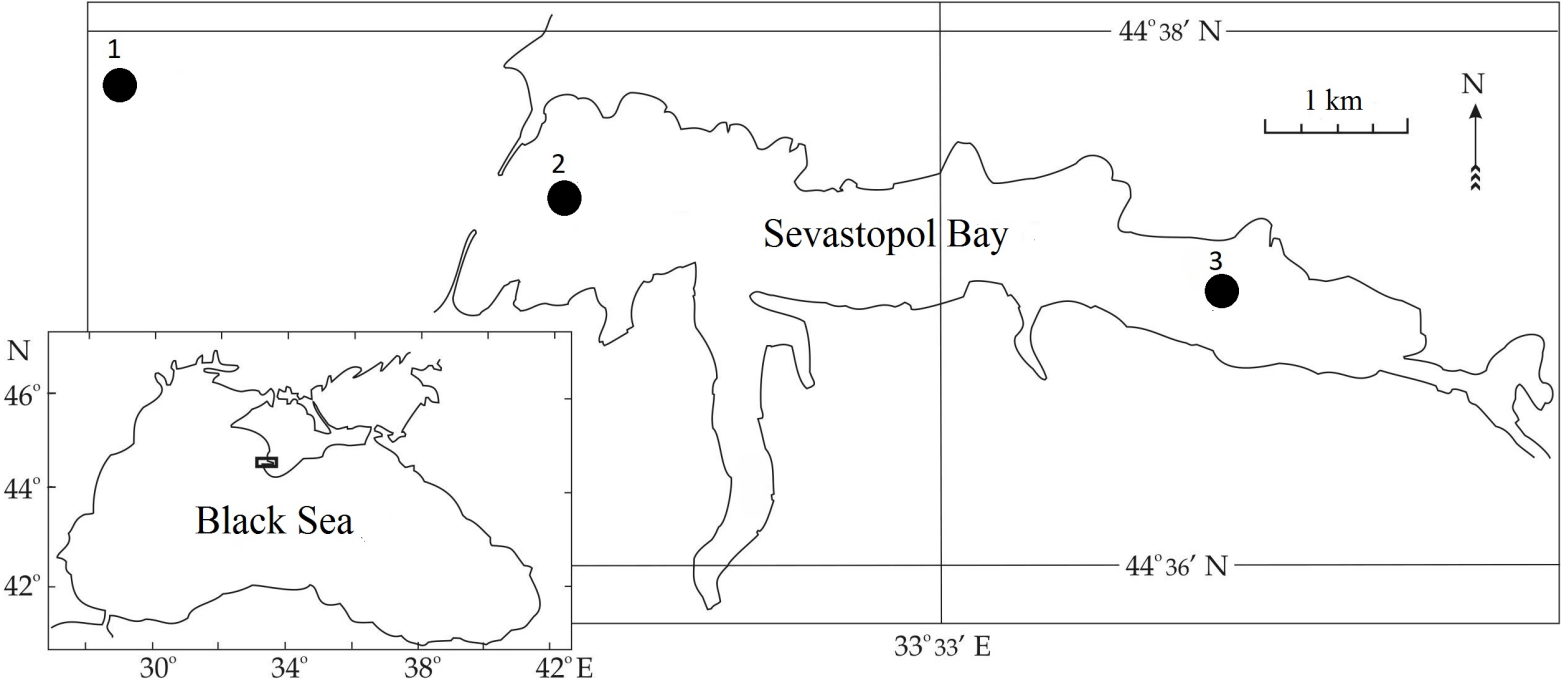
Sampling stations in Sevastopol Bay.

Sevastopol Bay is located in the northern part of the Black Sea at the southwestern tip of the Crimean Peninsula (Fig. 1). It is a semi-enclosed estuarine-type bay with restricted water exchange, is about 7 km long and 1 km wide at its widest point, and has an average depth of 12 m. According to long-term hydrological observations, Sevastopol Bay’s average sea surface temperature (SST) varies between 6 and 8 °C during winter and between 23 and 26 °C during summer (Garmashov, 2019). During the studied period, SST values stayed within this range of average values. The salinity values did not significantly change and were within the range of 17–18 ppt. The bay is a port area greatly affected by maritime traffic and anthropogenic factors, including NIS invasions. Regular investigations of the bay’s zooplankton were conducted in 1976, 1980, 1989, 1990, 1995, and 1996. They resumed in 2002 and have continued to this day.

Zooplankton samples were collected between 2016 and 2018 during routine surveys at three stations located within or adjacent to the Sevastopol Bay area (Fig. 1). In the bay, samples from Stations 2 and 3 (St. 2 and 3) were usually taken twice per month. Gaps occurred in May, July and October 2016, January and February 2017, and January, May, and September 2018 due to technical malfunction or meteorological conditions. Outside Sevastopol Bay, Station 1 (St. 1) samples were collected less frequently: in July, August, October, and December 2018. We collected samples using a Juday plankton net (with a mouth area of 0.1 m^2^ and a mesh size of 150 µm) from the whole water column: 40–0 m from St. 1, 10–0 m from St. 2, and 9–0 m at St. 3. Samples were collected in the morning and fixed with formaldehyde solution (4% final conc). They were processed in the laboratory using the methodology for zooplankton (Postel, Fock & Hagen, 2000; Aleksandrov et al., 2014). The *P. marinus* nauplii and copepodites were identified according to Uye & Onbe (1975).

We preserved the additional sample taken from St. 3 in December 2018 in 95% ethanol immediately after collection and stored it at 4 °C. Fifteen *P. marinus* individuals, CIV–CV, were extracted from the sample for genetic analysis.

### Morphological studies

We selected specimens for morphological study from zooplankton samples collected at Stations 2 (two females) and 3 (three females and three males) on October 8, 2018. The formalin-fixed specimens were immersed in a 1:1 solution of glycerin and water on glass slides, and were then measured and dissected. All operations were performed using a LOMO MBR-10 stereomicroscope. All line drawings were made using glycerine-mounted specimens and a camera lucida on a Leica DM LS2 compound microscope at magnifications 200 ×, 400 ×, and 1, 000 ×. We stained specimens with a solution of 1% chlorazol black E (SBE) dissolved in 70% ethanol for better visibility of the setae and aesthetascs.

Abbreviations used in the *P. marinus* drawings and descriptions were: Pr, prosome; Ur, urosome; Ur1–5, urosome somites; A1, first antenna (antennula); P5, fifth leg; Enp, endopod; Exp, exopod; and Exp1–3, exopod segments.

### Molecular genetic analysis

We obtained nucleotide sequences of the *cytb* gene fragment and nuclear ribosomal internal transcribed spacer (ITS1, 5.8 S, ITS2) using PCR-based sequencing procedures for *P. marinus* specimens.

*DNA extraction.* DNA was extracted using a DNK-EHKSTRAN Kit (Syntol, Moscow, Russia). We selected single specimens from the ethanol-preserved samples and incubated them overnight in 100 µL of lysis buffer (Syntol) with 5 µL of Syntol Proteinase K and 1 µL of 2-mercaptoethanol at 56 °C. After lysing, the specimens were crushed with a vortex pestle for 20 s and we carried out DNA extraction according to the DNK-EHKSTRAN Kit manufacturer’s protocol. The elution volume was 30 µL, and the DNA was stored at −20 °C.

*PCR amplification and sequencing*. The total 20 µL reaction mix volume for both DNA fragments was prepared as follows: 5xPCR Mix with MgCl_2_ (Evrogen, Moscow, Russia), 0.6 µM of each primer, and 2 µL of template DNA.

The *cytb* fragment was amplified using the primers UCYTB151F (5′-TGT GGR GCN ACY GTW ATY ACT AA-3′) and UCYTB270R (5′-AAN AGG AAR TAY CAY TCN GGY TG-3′) (Merritt et al., 1998). The reaction conditions included initial denaturation at 94 °C for 2 min, 35 cycles of denaturation at 94 °C for 30 s, annealing at 50 °C for 30 s, extension at 72 °C for 1 min, and a final extension at 72 °C for 4 min (Ohtsuka et al., 2018).

The ITS2 fragment was amplified using the primers ITS4 (5′-TCCTCCGCTTATTGATATGC-3′) and ITS1 (5′-TCCGTAGGTGAACCTGCGG-3′) (White et al., 1990). The reaction conditions included initial denaturation at 95 °C for 3 min, 40 cycles of denaturation at 95 °C for 30 s, annealing at 45 °C for 30 s, an extension at 72 °C for 30 s, and a final extension at 72 °C for 5 min.

We electrophoretically separated amplicons using a 1% agarose/TBE buffer gel with ethidium bromide, and visualised them using an ultraviolet (UV) transilluminator. PCR products were sequenced in both directions using the standard BigDye Terminator Cycle Sequencing Ready Reaction Kit (Applied Biosystems Inc., Foster City, CA, USA) and an ABI PRISM 3500xL analyser (Evrogen, Moscow, Russia).

*Sequence analysis.* We aligned the rDNA fragments using the BioEdit software program (Hall, 1999) and a *P. marinus* speciment from GenBank as a reference sequence for ITS2 (GenBank KT808252), and then we manually refined the alignment. The multiple alignment was run using ClustalW (Thompson, Higgins & Gibson, 1994) in MEGA7 (Kumar, Stecher & Tamura, 2016) software. We deposited all nucleotide sequences generated during this study in the GenBank database.

Evolutionary analysis was conducted using MEGA7. As shown earlier, ITS1 is more variable than ITS2 (Sabia et al., 2017) and is recommended for inter-population surveys, while ITS2 is usually used more for species discrimination. We decided to conduct phylogenetic analysis for both rRNA regions of ITS1 and ITS2. We determined the best substitution model using the Bayesian information criterion (BIC) and Akaike information criterion (AIC) model tests for best fit (Posada & Crandall, 2001; Raftery, 1995). The Kimura-2-parameter model and a discrete Gamma distribution (five categories, +G) were used for phylogenetic reconstruction using the maximum likelihood (ML) method (Kimura, 1980). We also determined the evolutionary distances inside the *Pseudodiaptomus* genus using the Kimura 2-parameter model (Kimura, 1980). The rate variation across sites was modeled with a Gamma distribution (shape parameter = 1). The analysis involved 10 *Pseudodiaptomus* species nucleotide sequences, and all positions with less than 95% site coverage were eliminated.

We aligned *cytb* sequences from our sample against sequences found in GenBank using BLAST (Altschul et al., 1990).

## Results

### Morphological examination

Description of female. Total body length 1.25–1.29 mm (mean 1.27 mm; *n* = 5).

**Figure 2:**
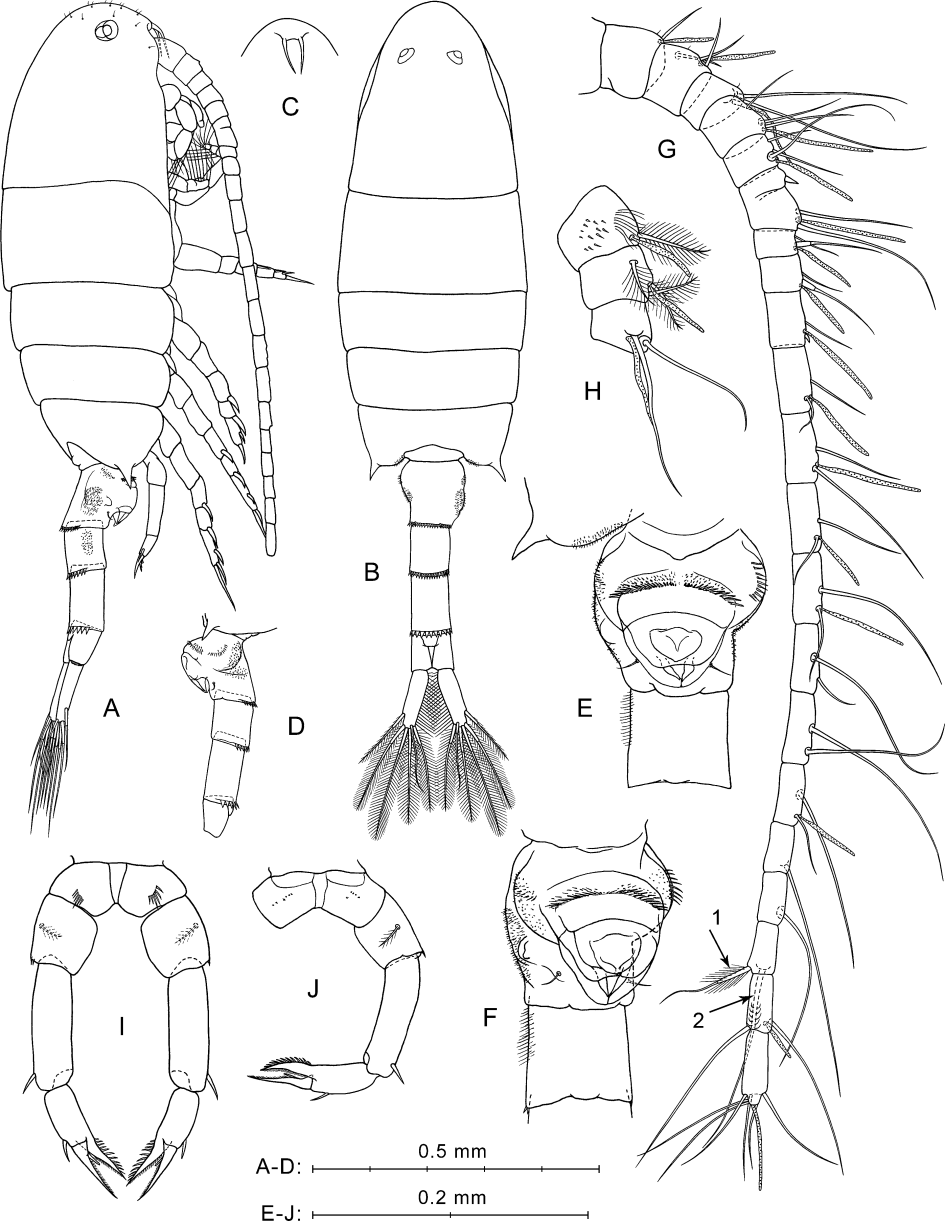
*P. marinus*Sato, 1913, female. (A) Lateral view. (B) Dorsal view. (C) Rostrum, ventral view. (D) Ur without caudal rami, left side. (E) Ur1–Ur2, ventral view. (F) Ur1–Ur2, latero-ventral view. (G) Right A1, dorsal view. (H) Left A1, segments 1–3, ventral view. (I) P5, anterior view. (J) Right P5 and left coxa, posterior view. Here and in Fig. 3, arrows indicate modified setae on A1 segment 20.

**Prosome** (Figs. 2A, 2B) elongated, about 2.5 times longer than wide, with broadly rounded anterior part in lateral view (Fig. 2A) covered with rare short hairs (not shown in Fig. 2B). Pedigerous somites 4 and 5 fused. Posterior corners produced into sharp spines directed outward. Rostrum with paired filaments (Fig. 2C). Eyes clearly visible, both in lateral and dorsal projections.

**Figure 3:**
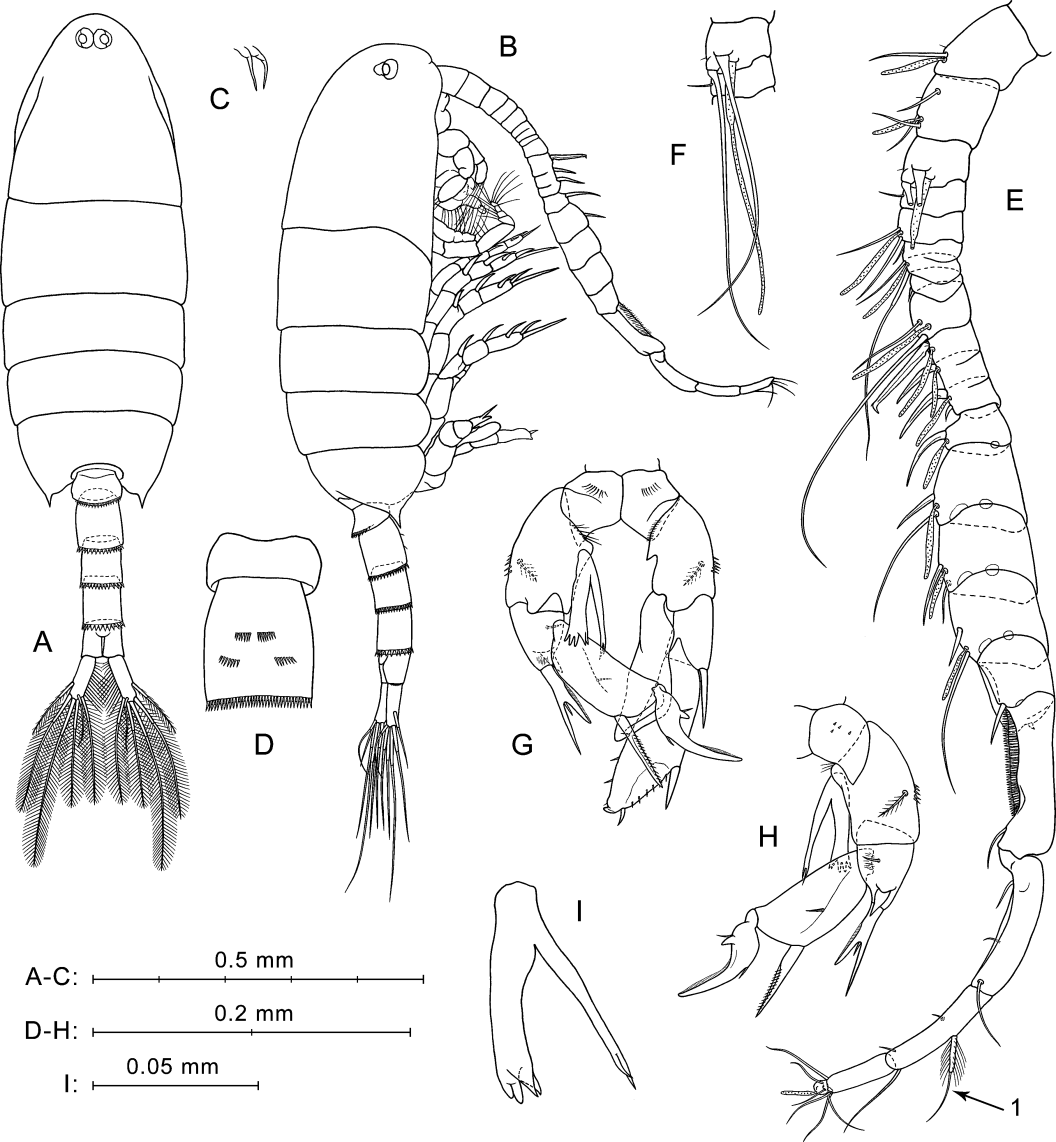
*P. marinus*Sato, 1913, male. (A) Dorsal view. (B) Lateral view. (C) Rostrum. (D) Ur1–Ur2, ventral view. (E) Right A1, ventral view. (F) Right A1, segments 3 and 4, ventral view. (G) P5, anterior view. (H) Right P5, posterior view. (I) Enp of right P5, anterior view, another specimen.

**Urosome** four-segmented (Figs. 2A, 2B, 2D). Pr/Ur about 1.8. Genital double-somite (Ur1) similar to that shown in a scanning electron micrograph by Soh et al. (2001: p. 213, Fig. 8B): asymmetrical, strongly swollen ventral side (Figs. 2A, 2D), lateral surfaces with asymmetrical patches of fine spinules, rows of stronger spinules on left lateral surface and proximal part of ventral protrusion, and one pair of thin setae near lower edge of ventral protrusion (Figs. 2E, 2F). Ur2 with rather long hairs perpendicular to right lateral surface (Figs. 2E, 2F). Ur3 longer than Ur2 and Ur4. Postero-dorsal margins of Ur1–Ur3 with rows of flat triangular teeth increasing in size from first to third somites (Figs. 2A, 2B, 2D).

**Caudal rami** symmetrical and diverge (Fig. 2B). Each ramus about three times longer than wide, with plumose inner side, and six setae (one lateral, one dorsal and four distal). All caudal setae plumose, except for dorsal seta with rare hairs. Longest caudal seta about 16% female total length.

**Antennulae** symmetrical, 22-segmented (segments 6 and 7 partly fused; counted separately), reaches about mid-length of Ur2 (Fig. 2A). Number and location of setae, spines, and aesthetascs on each segment shown in Fig. 2G and Table 1. First segment has groups of small spines and long hairs on ventral surface (Fig. 2H). Segments 1–4 bear tiny hair-like seta (Fig. 2G; not mentioned in Table 1). Segment 20 has two distal modified setae; shorter of them has thickened and elongated plumose base (Fig. 2G, arrow 1), and second seta with several pairs of recurved spines (Fig. 2G, arrow 2).

**Table 1:** Armature of the female and right male antennula.

Segment	Female	Male	Segment	Female	Male	Segment	Female	Male
1	1s+ae	1s+ae	9	2s+ae	2s+ae	17	2s+ae	1s+1sp
2	3s+ae	3s+ae	10^2^	1s+1sp+ae	1sp+ae	18	1s	1s
3	2s+ae	2s+ae	11^2^	2s+ae	1sp+ae	19	1s	3s
4	3s+ae	1s	12^2^	2s+ae	1sp+1ssp+ae	20	2s	4s
5	3s+ae	2s+ae	13	2s+ae	1sp+ae	21	2s+ae	
6^1^	1ssp	1s	14	2s+ae	1s+1sp+ae	22	6s+ae	6s+ae
7^1^	2s+ae	2s+ae	15	2s	1s+1sp+ae			
8	2s+ae	1ssp	16	2s	1s+1sp+ae			

**Notes.**

Segments partly fused: (1) in both sexes, (2) in male; s, seta; sp, ssp, spine, short spine; ae, aestethetasc.

**Fifth legs** symmetrical, uniramous (Figs. 2I, 2J). Coxa with row of spines on anterior mid-surface (Fig. 2I) and tiny spines in posterior view (Fig. 2J). Basis with medial plumose seta on posterior surface and one or two small spinules on distal outer corner. Exp three-segmented. Exp1 elongated, with one distal outer spine. Exp2 with one outer spine and well-developed pointed serrated process on inner side, which about equal in length to Exp2. Exp3 pointed, slightly longer than serrated process of Exp2, with small inner spiniform process near base.

Description of male. Total body length 1.05–1.11 mm (mean 1.08 mm; *n* = 3).

**Prosome** (Figs. 3A, 3B) similar to that of female, but slightly more slender, about 2.6 times longer than wide. Posterior corner spines directed backwards. Rostrum with paired filaments (Fig. 3C). Eyes located close to each other (Fig. 3A).

**Urosome** five-segmented (Figs. 3A, 3B). Pr/Ur about 2.0. Distal margins of Ur1–Ur4 with rows of flat triangular teeth similar to those in female, but around the entire perimeter of somite, excluding Ur1 (teeth only on dorsal margin). Size of teeth increases from Ur1 to Ur4. Ur2 with two paired groups of small spines on ventral surface (Fig. 3D).

**Caudal rami** (Fig. 3A) similar to those of female, but most caudal setae relatively longer. Longest seta about 25% male total length.

**Antennulae** asymmetrical. Left A1 similar to that of female (not shown in Fig. 3). Right A1 geniculate (between segments 18 and 19), 21-segmented (segments 6–7 and 10–12 appear to be partly fused; counted separately) (Figs. 3E, 3F; Table 1). Ventral surface of first segment has no small spines or long hairs (Fig. 3E). Segments 1–3 have tiny hair-like seta (not mentioned in Table 1). Segment 10 has a large hooked spine. Segment 20 with two pairs of setae, distal and medial; one seta from each pair strongly shortened, and long medial seta (Fig. 3E, arrow 1) similar to shorter modified seta on segment 20 of female A1 (see Fig. 2G, arrow 1).

**Fifth legs** (Figs. 3G, 3H) strongly asymmetrical with well-developed endopods on both left and right P5. Coxa similar to that of female, with row of spines on anterior surface (Fig. 3G) and tiny spines in posterior view (Fig. 3H). Right basis larger than left, with row of thin spinules on proximal margin in anterior view. Left basis with fine proximal hairs in anterior view, and with short pointed protrusion proximally on inner lateral surface. Both right and left basal segments with medial plumose setae on posterior sides and with rows of spines on outer lateral surfaces.

Left Exp (Fig. 3G) bi-segmented. Exp1 short, with one strong spine on outer distal corner. Exp2 elongated, narrowed distally, with three thin spinules on distal half of inner margin, and two spines on outer margin (lateral spine much larger than distal one); spinulous margin between these spines.

Left End uni-segmented (Fig. 3G), elongated, lob-shaped, narrowed distally, reaches more than mid-length of Exp2.

Right Exp (Figs. 3G, 3H) three-segmented. Exp1 has stout distolateral spine forked near mid-length with small process between rami; outer ramus longer and thinner than inner. In posterior view (Fig. 3H), Exp1 has short thick spine near base of forked spine, small medial seta, and patch of thin hairs. Exp2 elongated, with long, straight, partly serrated spine on outer distal corner and small medial seta on posterior surface. Exp3 has long sickle-shaped outer process, short inner process, and two small setae near their bases.

Right Enp (Figs. 3G, 3H) has two rami extending beyond distal edge of right basis. One of them bifurcated at apex, slender, and slightly longer than another ramus. Thicker ramus ends with four to five spiniform processes (Figs. 3G, 3I), two of which sharp-pointed and others with slightly rounded tips. Two pointed processes may seem fused into one depending on the angle of view.

### Molecular genetic analysis

The rRNA sequences, including the ITS1, 5.8S, and ITS2 regions obtained from most of the individuals collected from the Black Sea, revealed a notable intraindividual heterogeneity observed using the electropherograms obtained by direct and reverse sequencing. We detected the presence of several allelic variants by double peaks. Because divergent rRNA copies affect phylogenetic reconstruction (Karlep, Reintamm & Kelve, 2013), we excluded all heterogenetic sequences from the analysis. Only three sequences had one allelic variant, and these sequences were deposited in GenBank (accession numbers MN555816–MN555818). The length of these sequences varied: PMB7 –  706 bp (ITS1, 5.8S, ITS2), PMB11 –  416 bp (ITS2), and PMB14 –  414 bp (ITS2).

**Figure 4:**
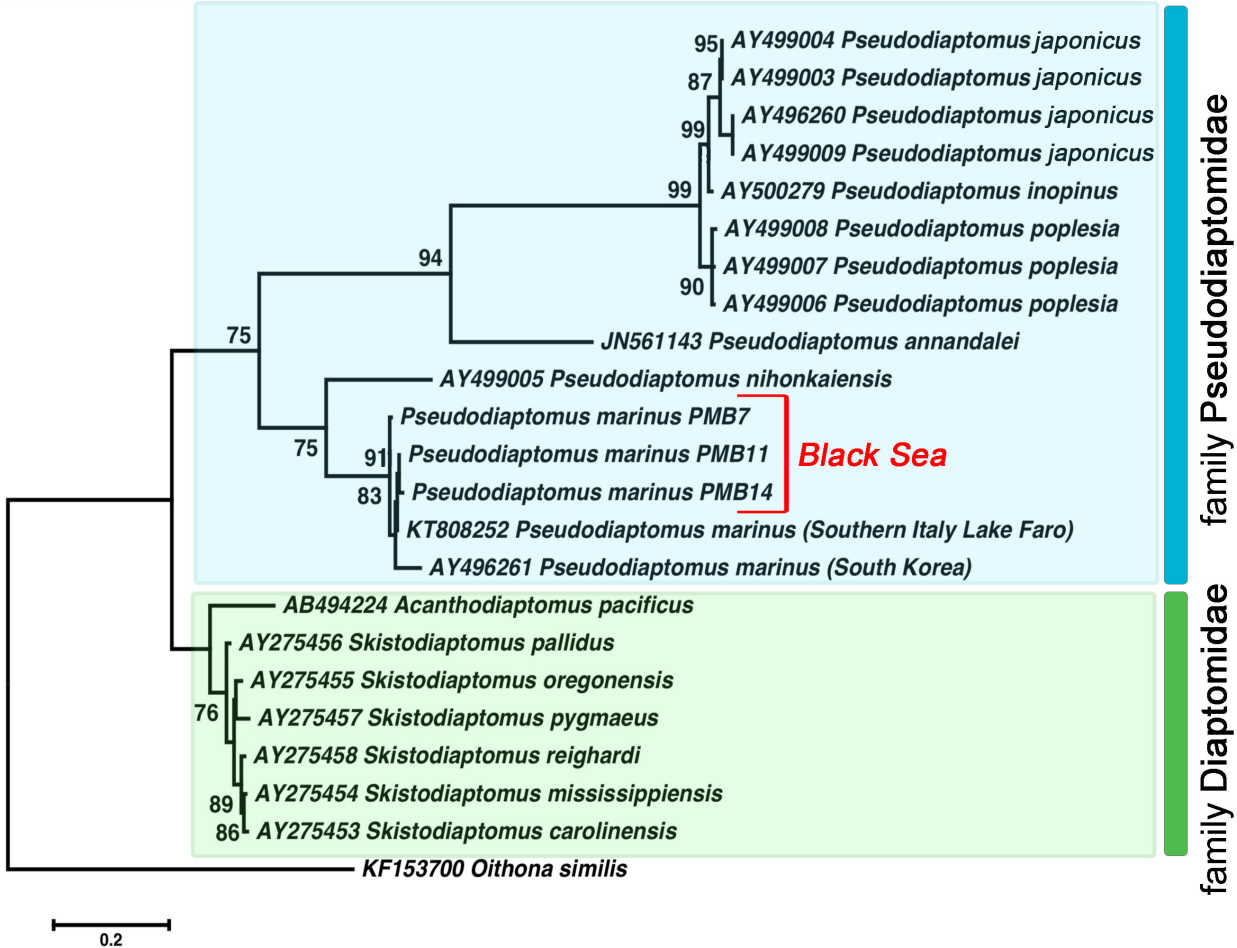
The phylogenetic tree constructed on the basis of 22 nucleotide sequences of nuclear ribosomal internal transcribed spacer (ITS2) by ML method (K2 model and a discrete Gamma distribution). The indices of bootstrap analysis with values higher than 75% are shown. *P. japonicus* (AY496260, AY499003, AY499004, AY499009) is presented as *P. koreanus* Soh et al., 2012 in the Genbank database. The last was recently relegated as a junior synonym of *P. japonicus* Kikuchi, 1928 (Sakaguchi & Ueda, 2018).

During our calculations, we treated the beginning of the rRNA sequences (which did not have information about ITS1) as missing data. The total length of alignment was 742 bp. We conducted the rDNA-based phylogeny with some Pseudodiaptomidae and Diaptomidae species sequences from GenBank (their accession numbers are presented in Fig. 4), and we used *Pseudodiaptomus* specimens from the Black Sea and cyclopoid copepod *Oithona similis* (KF153700) as the outgroups.

All *Pseudodiaptomus* individuals collected from the Black Sea were grouped with two other *P. marinus* individuals in a separate clade (ML, 91%) found in Genbank. We identified relatively high genetic heterogeneity within this species. The phylogenetic tree demonstrated 3% divergence between individuals from South Korea and individuals from the Black Sea and Lake Faro (in the Mediterranean basin). The genetic distance within the *P. marinus* species was the same as the distance between different *Pseudodiaptomus* species (Table 2, shown in bold).

**Table 2:** Estimates of evolutionary divergence between *Pseudodiaptomus* species. The number of base substitutions per site from between sequences are shown. Standard error estimate (s) are shown above the diagonal.

		1	2	3	4	5	6	7	8	9	10
1	*P. marinus* PMB7		0.008	0.008	0.012	0.007	0.028	0.061	0.087	0.085	0.081
2	*P. marinus* PMB14	0.017		0	0.011	0.003	0.029	0.057	0.084	0.085	0.082
3	*P . marinus* PMB11	0.017	0		0.011	0.003	0.029	0.057	0.084	0.085	0.082
4	*P. marinus* (South Korea)	**0.043**	**0.032**	**0.032**		0.011	0.03	0.057	0.082	0.083	0.079
5	*P. marinus* (Southern Italy Lake Faro)	0.014	0.003	0.003	**0.032**		0.029	0.057	0.084	0.085	0.082
6	*P. nihonkaiensis*	0.157	0.162	0.162	0.173	0.167		0.076	0.076	0.075	0.078
7	*P. annandalei*	0.398	0.374	0.374	0.372	0.374	0.484		0.053	0.054	0.053
8	*P . japonicus*	0.548	0.533	0.533	0.522	0.533	0.486	0.353		0.009	0.011
9	*P . inopinus*	0.536	0.533	0.533	0.522	0.533	0.476	0.353	**0.021**		0.01
10	*P . poplesia*	0.515	0.513	0.513	0.501	0.513	0.489	0.345	**0.036**	**0.028**	

**Notes.**

Values, highlighted in bold and in red, indicate minimum genetic divergence thresholds of species groups (see text for details).

We obtained fifteen sequenced fragments for the mitochondrial *cytb* gene with lengths ranging from 344 to 374 bp and deposited them in the GenBank (accession numbers MN561264–MN561278). Based on the blast similarities in GenBank, we identified all haplotypes as *P. marinus (Ohtsuka et al., 2018)*. Three haplotypes were found in a total of 15 *cytb* sequences from the Black Sea: 10 individuals demonstrated 99.73% identity (100% query) with AB920868, three individuals demonstrated 99.73% identity (100% query) with AB920869, and we found 99.73% identity (100% query) with AB920872 in two individuals. These sequences corresponded to the H1, H2, and H5 haplotypes (Ohtsuka et al., 2018). All haplotypes were shared between Japanese coastal waters and the San Francisco Estuary, and only two (H1 and H2) were detected from Haeui Island, Korea.

### Seasonal and interannual variations in abundance

**Figure 5:**
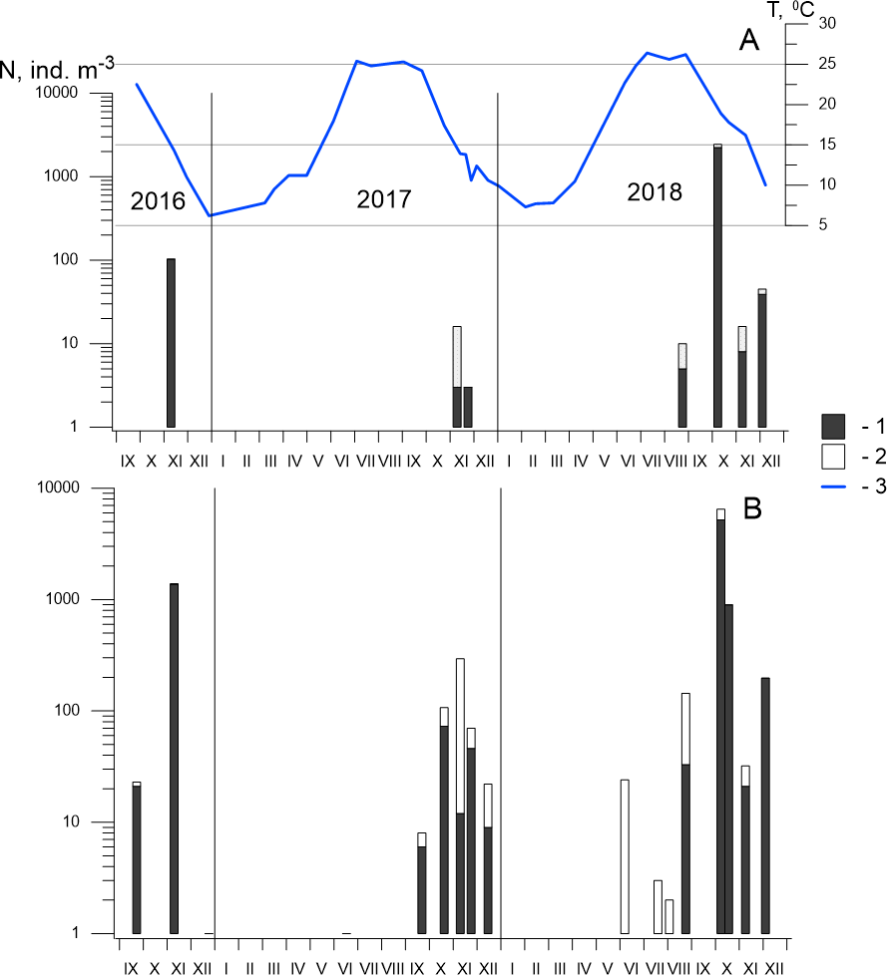
Seasonal and interannual variations in abundance of *P. marinus* in Sevastopol Bay. (A) at the station 2; (B) at the station 3. 1, CI–CVI copepodite development stages including females and males (CVI); 2, nauplii; 3, temperature. Although the size of the nauplii was 0.21–0.24 mm in the samples and their abundance is underestimated in our net with mesh size 150 µm, we included them in the dataset because of the high abundance throughout the entire study period, which indicates the reproduction of species in the Sevastopol Bay.

*P. marinus* was first found in the Black Sea during a routine survey of Sevastopol Bay at the end of September 2016. We recorded 23 ind m^−3^ of this alien species at each developmental stage, including six females at the centre of the bay (St. 3) and only two nauplii at its mouth (St. 2). In November 2016, the abundance of *P. marinus* had increased significantly, reaching up to 1,373 ind m^−3^ at St. 3 and 103 ind m^−3^ at St. 2 (Fig. 5), which were the highest densities that year. The following month, the population abundance had decreased sharply, and only one female was found at St. 3.

In 2017, *P. marinus* was not found until September, except for one young copepodite found in June at St. 3 (Fig. 5). The abundance was low (6 ind m^−3^) in September and increased to 73 ind m^−3^ in October, which was significantly lower than the density in October 2016, which saw the highest recorded abundance. Therefore, we collected samples every week in November 2017 to determine the annual population maximum. However, the abundance varied between 12 and 46 ind m^−3^ and did not exceed the levels measured in October. In December, the abundance decreased to 9 ind m^−3^.

In 2018, *P. marinus* was not found in samples during the first half of the year, similarly to 2017. Single copepodites appeared in June and were found in July and early August at both stations. We observed a small rise in abundance at the end of August (33 ind m^−3^) at St. 3. The peak in abundance was recorded in early October when the density increased to 5,184 ind m^−3^ at St. 3 and 2,157 ind m^−3^ at St. 2, which was the highest *P. marinus* abundance during the whole research period. Copepodites, nauplii, and adults of both sexes, including egg-carrying females, were found in this sample. Additionally, males were recorded for the first time in the studied period. In November and December, the abundance dropped down to 21 ind m^−3^ and 197 ind m^−3^ at St. 3, and 8 ind m^−3^ and 39 ind m^−3^, at St. 2 , respectively.

Altogether, we found *P. marinus* in 12 of the 26 samples collected from the centre of Sevastopol Bay (St. 2), and in 19 of the 26 samples collected at its mouth (St. 3). Outside the bay (St. 1), *P. marinus* was not found during the entire studied period.

Our year-round observations of *P. marinus* in Sevastopol Bay during 2017–2018 showed that it did not appear during the first half of the year in the pelagic community (Fig. 5). Single young copepodites and nauplii appeared in June when the temperature reached 21−22 °C. We recorded considerable levels from September to the end of the year, with the maximum abundance recorded in October. The water temperature was 17.3 °C in 2017 and 19.6 °C in 2018, which were lower than the optimum temperature (20−25 °C) for this species’ egg production (Uye & Kasahara, 1983). However, favourable temperatures (20−22 °C) were recorded two weeks earlier (from the 20th of September to the 6th of October) that could support intensive population growth. The development of *P. marinus* from eggs to adults was shown to be 13 days (Huang, Zhu & Liu, 2006) so the species abundance can rapidly increase under the favourable conditions (Huang, Zhu & Liu, 2006; Deschutter et al., 2018).

## Discussion

### Taxonomic identification and variability; vector of introduction

*P. marinus* belongs to the Ramosus species group and *hickmani*-subgroup, which include nine morphologically close species (Walter, Ohtsuka & Castillo, 2006). *P. marinus* is distinct from these species because of its combination of the following features (based mostly on the structural details of male P5):

(a) distolateral spine on right Exp1 bifurcated near mid-length, with the outer ramus longer and thinner than the inner ramus (typical only for *P. marinus*);

(b) left Enp narrowed distally (not rounded);

(c) well-developed right Enp with two rami extending beyond the distal edge of right basis, thicker ramus ends with three to six spiniform processes.

**Figure 6:**
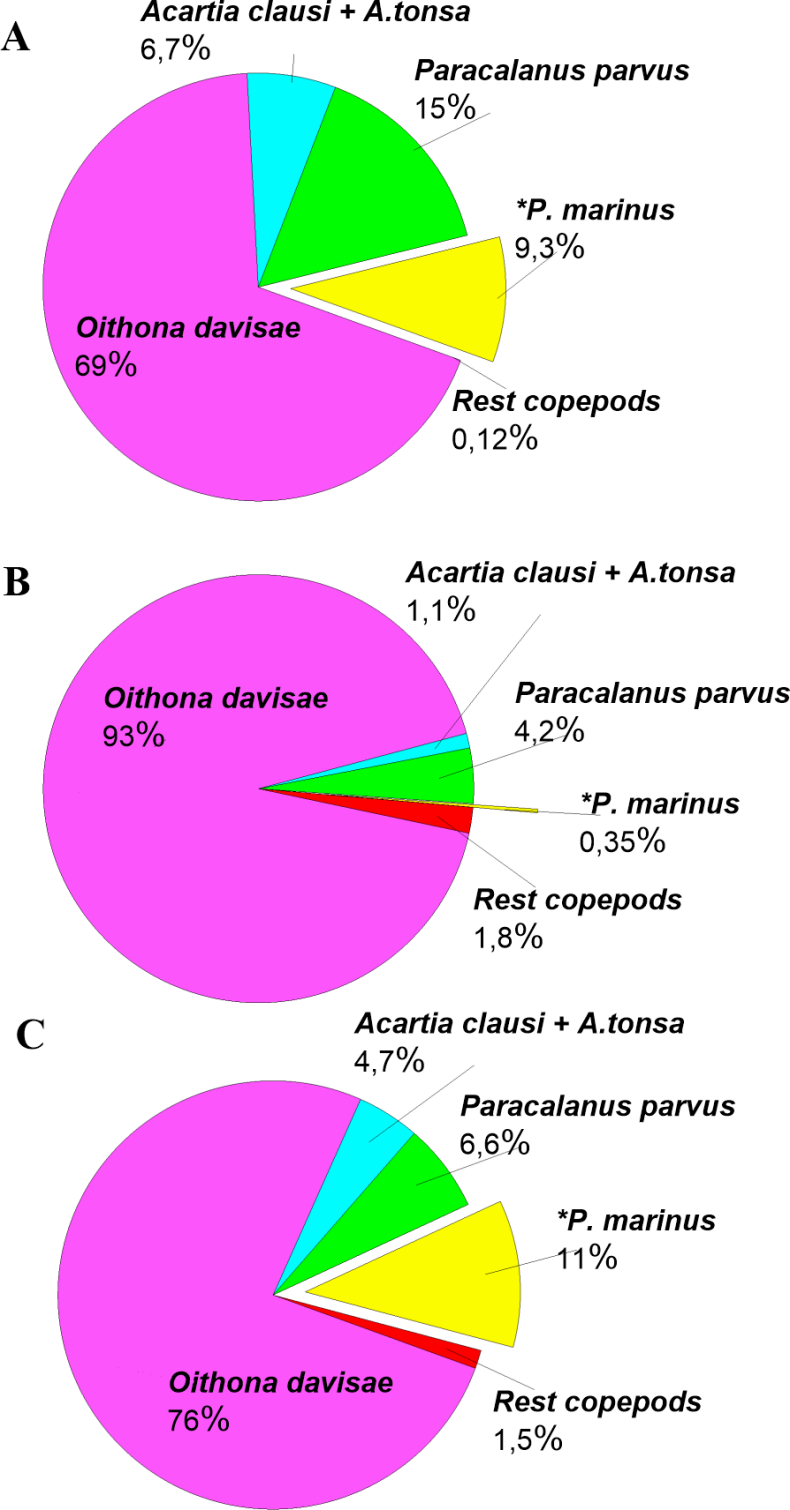
Copepods species percentage in central part of Sevastopol Bay (St. 3) during *P. marinus* abundance peaks in 2016–2018. (A) 2016/11/09; (B) 2017/10/19; (C) 2018/10/08. Rest copepod group includes *Oithona similis*, *Pseudocalanus elongatus*, *Calanus euxinus* and *Centropages ponticus*. Most abundant species are *Oithona davisae, Paracalanus parvus, Acartia clausi+A.tonsa, P. marinus*.

The drawings and descriptions of *P. marinus* can be found in Sato (1913: pl. 7, Figs. 69–71), Brodskii (1948: p. 63–64, 96, 116, Table 18, Figs. 1–7), Brodskii (1950: p. 322, Fig. 225), Tanaka (1966: p. 44, Fig. 4 (from Razouls et al., 2005–2020)), Kos (1985: p. 236, Figs. 6, 7), Nishida (1985: p. 132, Fig. 4f–j), Walter (1986: p. 146–147, Fig. 7L–O), Fleminger & Kramer (1988: p. 539–540, Fig. 4), Soh et al. (2001: p. 213, Fig. 8B), and Brylinski et al. (2012: p. 580, Fig. 3). In contrast, specimens from the coastal waters of the Indian Ocean (Grindley & Grice, 1969; Pillai, 1976) and eastern Australia (Greenwood, 1977) are not *P. marinus*, but distinct and closely related species (Walter, 1986; Walter, 1987; Fleminger & Kramer, 1988).

After comparing diagnostic features, we observed that *Pseudodiaptomus* specimens from the Black Sea resembled *P. marinus* Sato, 1913. Some differences, namely the perpendicular hairs on the right lateral surface of Ur2, a group of long hairs on the ventral surface of the first A1 segment in females, and a modified seta with a thickened plumose base on segment 20 of the right A1 in males, are probably not specific to Black Sea specimens but have not been previously recorded. However, we did note the variability in the number of spiniform processes at the end of the P5 right Enp in Black Sea males. Of the three examined males, two specimens had four processes (see Figs. 3G, 3H) and one specimen had five processes (see Fig. 3I). Walter (1986) re-examined individuals from various locations across Japan (including Nemuro Bay, Hokkaido, near the type locality of *P. marinus*) and from Ala Wai Canal, Hawaii, and found connections between the number of these processes and the geographical location of the re-examined individuals. Fleminger & Kramer (1988) found a similar variation to Walter’s (1986), but in individuals from the same bay (Mission Bay, Southern California). They concluded that “these variations appear to vary randomly and may not characterise geographically different stocks within the species” (Fleminger & Kramer, 1988: p 539). Our results were similar to those of Fleminger & Kramer (1988). However, further morphological studies are required for a clearer understanding of this phenomenon and the variability of other distinctive *P. marinus* features.

Currently, molecular genetic analysis is one of the most efficient ways to identify and confirm new invasive species, their region of origin, and possible dispersal pathways. Using the genetic variability of ITS2 and *cytb*, we determined that all analysed copepods from Sevastopol Bay belonged to the *P. marinus* species.

Moreover, our results revealed intraspecific genetic differentiation for *P. marinus*. Sabia et al. (2017) recorded the first evidence of such divergence and suggested that a ‘new form’ had inhabited Lake Faro, but no related studies followed. Our ribosomal ITS2 sequences showed small differences between Lake Faro and Black Sea specimens (a genetic distance of about 1.1% only), which allowed us to consider that these samples belonged to one group. At the same time, the ITS2 sequence from South Korea differed by 3.2–4.3%. The nucleotide diversity within *P. marinus* from South Korea is incompletely known due to sampling limitations. In copepods, ITS2 is considered less variable than ITS1 or COI (Makino, Knox & Duggan, 2010; Sabia et al., 2017) and the degree of interspecific differences varies from <1% to 55% for this marker. For the Eucalanidae family, no variation has been observed within species and only 0.2–3.4% divergence has been detected between the closest species (Goetze, 2003). We observed the same situation for the Diaptomidae family, in which phylogenetic reconstruction revealed small genetic interspecies differentiation (Fig. 4). Our interspecific genetic distance analysis for the *Pseudodiaptomus* genus showed an average divergence levels as high as 55% (Table 2). However, the *P. japonicus*, *P. inopinus*, and *P. poplesia* species group differed by 2.1–3.6%. (Table 2, shown in bold), which was even lower than the difference across *P. marinus* geographical groups. We confirmed the genetic differences for these three species using ITS1 (12–14%) and COI (17.6–26.7%) (Eyun et al., 2007; Soh et al., 2012). Therefore, closely related or recently diverged species may have quite low genetic differentiation that ranges between 2% and 5% at ITS2, making it likely that we found two cryptic species with 3.2–4.3% divergence (Table 2, shown in bold red).

When observing the ITS2 nucleotide diversity, we expected that individuals from Lake Faro and the Black Sea formed one group (the Mediterranean group) that differed significantly from the South Korean specimens. Low genetic distance values may be associated with the resolution of the molecular marker discussed above. We could not deduce the origin of the Mediterranean group from our genetic data because of insufficient information about ITS2 genetic diversity. Therefore, an increased number of samples from different parts of the distribution range and more variable molecular markers are required.

Previous studies have analysed the genetic and haplotype diversity of *P. marinus* populations from the coastal regions of Japan, Korea, and the San Francisco Estuary based on mtDNA *cytb*-sequenced data (Ohtsuka et al., 2018). Of the 39 haplotypes detected, three (H1, H2, and H5) accounted for more than half of the population in six of the seven studied localities. The only exception was Haeui Island, Korea, where 11 haplotypes were apparently endemic and only two (H1 and H2) were shared between the Korean, Japanese, and San Francisco Estuary localities. However, these haplotypes had a low frequency in Haeui Island that was different than other areas where they made up a significant part of the *P. marinus* population. Thus cytb sequence investigations, like the ITS2 ones sited above, reveal the specificity of the *P. marinus* population from Korean region. This fact allow to assume that another form (or cryptic species) is located just in South Korean waters not in Lake Faro as Sabia and coauthors have supposed (Sabia et al., 2017) and the Mediterranean *P. marinus* groups thus may be close to the population from other regions of the Western Pacific.

The same ubiquitous haplotypes H1, H2, and H5 (Ohtsuka et al., 2018) were found in the Black Sea at frequencies of approximately 67, 13, and 20%, respectively. The Black Sea had the lowest number of haplotypes of the studied localities (Ohtsuka et al., 2018). The low haplotype diversity of Sevastopol Bay *P. marinus* was consistent with the general pattern of genetic depletion in a newly introduced population, which is usually associated with strong genetic drift or bottleneck effects (Geburzi & McCarthy, 2018).

Using mtDNA *cytb* diversity analysis, Ohtsuka et al. (2018) confirmed that there were multiple *P. marinus* introductions in the San Francisco Estuary in the 1990s from several Japanese localities, although introductions from Korea could not be excluded. *P. marinus* was most likely introduced into the Black Sea from the Mediterranean Sea, where it had widely spread during the previous decade, and from where vessels had most often came into Sevastopol Bay. However, mtDNA *cytb* sequences for *P. marinus* populations in Mediterranean or South Atlantic waters cannot be found in the NCBI. It is currently impossible to conduct reliable genetic analysis to determine the donor region of the *P. marinus* population in the Black Sea.

Ballast water from trans-oceanic ships have been suggested as the main vector of *P. marinus* introduction into European coastal marine environments (Lučić et al., 2015; Sabia et al., 2015; Deschutter et al., 2018; Uttieri et al., 2020). Another suggested distribution route for this copepod is aquaculture (Zagami et al., 2018; Sabia et al., 2014), although there are no aquaculture farms inside Sevastopol Bay. It is most likely that *P. marinus* was introduced into the Black Sea via ballast water from ships, similarly to other non-indigenous copepods *Acartia tonsa* and *O. davisae* (Gubanova, 2000; Altukhov, Gubanova & Mukhanov, 2014), since *P. marinus* was first found in Sevastopol Bay and did not spread elsewhere until at least the end of 2018. The previous invader, *O. davisae,* was also initially found within the bay and began to spread to other coastal areas of the Black Sea four years later.

### Main causes of successful spread and establishment

*P. marinus* has been found in more than 10 countries (Uttieri et al., 2020; Suzuki, Nakayama & Tanaka, 2013). Great environmental adaptability and some biological features have allowed this species to survive in new areas. This copepod thrives in habitats with temperatures ranging from 6.3 to 31.5 °C and 2.5 to 38.5 ppt salinity (Sabia et al., 2015; Uttieri et al., 2020). According to an experimental study by Svetlichny et al. (2019), the adult *P. marinus* has a salinity tolerance ranging between 5.0 and 44.0 ppt, and a temperature tolerance ranging from 8.0 °C and 27.0 °C. *P. marinus* is epibenthic during the day, but swims up the water column at dusk and acts as a pelagic species during the night (Uye & Kasahara, 1983; Sabia et al., 2015). Therefore, both the pelagic and benthic feeding modes are available to *P. marinus*, and copepod can act as either herbivores or detritivores, respectively (Uye & Kasahara, 1983; Sabia et al., 2015). The species’ ecology and biological plasticity allow it to adapt to the Black Sea’s low salinity and low winter water temperatures and successfully compete (or avoid competition) with native species. Between 2016 and 2018, the salinity in Sevastopol Bay ranged from 17 to 18 ppt, and the temperature ranged from 6.2 to 26.2 °C. The lowest temperature was reported in December 2016 and the highest in August 2018.

Normally, the invasion of a new species is preceded by a series of changes in an ecosystem (Alimov et al., 2004). The imbalance of the Black Sea ecosystem was initially caused by eutrophication, pollution, and overfishing in the 1970s and 1980s. In turn, this preconditioned the invasion of predatory ctenophores in the 1990 and 2000s, which resulted in profound changes in the basin ecosystem (Shiganova et al., 2001; Gubanova et al., 2001; Kideys, 2002; Finenko & Romanova, 2000; Oguz, Fach & Salihoglu, 2008; Gubanova et al., 2019).

### Seasonal dynamics in the new environment

Previous studies on *P. marinus’* seasonal and interannual variability in abundance have shown that, since its first appearance in 2016, *P. marinus* has successfully survived in the new environment of Sevastopol Bay. We collected this species at all stages of its development, including ovigerous females and nauplii within the studied period of 2016 to 2018 (Fig. 5). Therefore, we concluded that the new invasive species reproduced there and established a self-sustaining population. We also observed identical seasonal patterns with one pronounced abundance peak in the autumn during 2017 and 2018. However, the abundance was noticeably higher in 2018 where we observed the species in plankton from July to December, while in 2017, the species was not found until September, with the exception of one individual in June, Our findings most concern St. 3, which was located in the centre of the bay. In the mouth of the bay at St. 2, *P. marinus* appeared more rarely, particularly during 2017, and with a lower density in comparison to St. 3. However, the frequency of *P. marinus* occurrence and abundance increased in 2018 at both stations in the bay. At the same time, we did not find specimens outside the bay throughout the studied period, although it is possible that *P. marinus* may spread outside the bay in the following year, similarly to *O. davisae,* another non-indigenous copepod (Altukhov, Gubanova & Mukhanov, 2014).

The density of *P. marinus* was mostly insignificant during the observed years. There were exceptions during the autumn peaks when its abundance reached 1,373 ind m^−3^ (in 2016), 73 ind m^−3^ (in 2017), and 5,183 ind m^−3^ in 2018 (the highest one). *O. davisae* was a dominating species in the copepod community, representing 70–90% of total copepod abundance along with *Paracalanus parvus*, *Acartia clausi,* and *A. tonsa* (Fig. 6). This has been the typical composition of copepod species for autumn in Sevastopol Bay since 2006 (Gubanova et al., 2019). The new member of the community, *P. marinus,* did not significantly affect the species ratio and made up less than 1% of the copepod community, except during its maximum abundance in autumn 2016 and 2018 when its percentage amounted to 9 and 11%, respectively, and exceeded that of the *Acartia* species (Fig. 6).

*P. marinus* is reported as a rare species at most sites in the Mediterranean Sea. Its abundance is usually lower than 200 ind m^−3^ and can even be lower than 20 ind m^−3^ (Uttieri et al., 2020). Sevastopol Bay’s abundance of more than 5,000 ind m^−3^ is one of the highest in the Mediterranean basin. However, according to our data, *P. marinus* seasonal dynamics are characterised by one very sharp increase in abundance that lasts for a short period of time. Regular and frequent sampling should be conducted to catch such short-term increase in the species’ number.

*O. davisae* has occupied a free ecological niche and has replaced *O. nana* in the Black Sea, while *A. tonsa* has ousted *Acartia latisetosa* due to a number of competitive advantages (Gubanova, 2000; Gubanova et al., 2014). Since *P. marinus* is not as abundant yet, its total effect on the native copepod community has not been revealed. *P. marinus*’ ability to switch between pelagic (night) and demersal (day) life modes may provide it with competitive advantages in the Black Sea community. However, this hypothesis has to be thoroughly tested in future investigations.

## Conclusions

Additional studies on the *P. marinus* invasion in the Black Sea are necessary to fully understand this alien species’ introduction into a new habitat with specific environmental conditions (low salinity, low winter temperature, and low species diversity) and the influence of the new invader on the recipient community in comparison to other European sites. The successful establishment of *P. marinus* into new ecosystems with different environmental conditions and anthropogenic influences may also lead to morphological and genetic variations. Therefore, future studies on *P. marinus’* morphological characteristics across different sites, combined with genetic analysis, are still crucial. These challenges are currently being addressed through international cooperation and the EUROBUS working group.

##  Supplemental Information

10.7717/peerj.10153/supp-1 Supplemental Information 1 DNA sequences:  MN561264 to  MN561278

10.7717/peerj.10153/supp-2 Supplemental Information 2 Raw data on *P. marinus* sampling in the Black Sea (Sevasopol Bay) The file includes columns with the following information: (1) Year; (2) Month; (3) Day of sampling; (4) station number ; (5) - (6) depth of sampling - from and up to correspondingly; (7) volume of filtered water; (8) stage of development, where: f- female, m –male, C1-5 - copepodite development stages, n - nauplii, nf - not found; (9) ind/sample - abundunce P. marinus in the sample; (10) ind/m3 - abundunce P. marinus in m3; (11) T ° - sea surface temperature.

## Additional Information and Declarations

### DNA Deposition

The following information was supplied regarding the deposition of DNA sequences: The sequenced fragments for mitochondrial cytb gene are available at GenBank: MN561264 to MN561278. The rRNA sequences ITS1, 5.8S, ITS2 are available at GenBank: MN555816, MN555817 to MN555818.

### Data Availability

The following information was supplied regarding data availability: The raw measurements are available as a Supplemental File.
